# Endovascular Management of Post-Irradiated Carotid Blowout Syndrome

**DOI:** 10.1371/journal.pone.0139821

**Published:** 2015-10-06

**Authors:** Feng-Chi Chang, Chao-Bao Luo, Jiing-Feng Lirng, Chung-Jung Lin, Han-Jui Lee, Chih-Chun Wu, Sheng-Che Hung, Wan-Yuo Guo

**Affiliations:** 1 Department of Radiology, Taipei Veterans General Hospital, Taipei, Taiwan; 2 National Yang Ming University, School of Medicine, Taipei, Taiwan; University of Pennsylvania School of Medicine, UNITED STATES

## Abstract

**Purpose:**

To retrospectively evaluate the clinical and technical factors related to the outcomes of endovascular management in patients with head-and-neck cancers associated with post-irradiated carotid blowout syndrome (PCBS).

**Materials and Methods:**

Between 2000 and 2013, 96 patients with PCBS underwent endovascular management. The 40 patients with the pathological lesions located in the external carotid artery were classified as group 1 and were treated with embolization. The other 56 patients with the pathological lesions located in the trunk of the carotid artery were divided into 2 groups as follows: group 2A comprised the 38 patients treated with embolization, and group 2B comprised the 18 patients treated with stent-graft placement. Fisher’s exact test was used to examine endovascular methods, clinical severities, and postprocedural clinical diseases as predictors of outcomes.

**Results:**

Technical success and immediate hemostasis were achieved in all patients. The results according to endovascular methods (group 1 vs 2A vs 2B) were as follows: technical complication (1/40[2.5%] vs 9/38[23.7%] vs 9/18[50.0%], *P* = 0.0001); rebleeding (14/40[35.0%] vs 5/38[13.2%] vs 7/18[38.9%]), *P* = 0.0435). The results according to clinical severity (acute vs ongoing PCBS) were as follows: technical complication (15/47[31.9%] vs 4/49[8.2%], *P* = 0.0035); rebleeding (18/47[38.3%] vs 8/49[16.3%], *P* = 0.0155). The results according to post-procedural clinical disease (regressive vs progressive change) were as follows: alive (14/21[66.7%] vs 8/75[10.7%], *P<*0.0001); survival time (34.1±30.6[0.3–110] vs 3.6±4.0[0.07–22] months, *P*<0.0001).

**Conclusion:**

The outcomes of endovascular management of PCBS can be improved by taking embolization as a prior way of treatment, performing endovascular intervention in slight clinical severity and aggressive management of the post-procedural clinical disease.

## Introduction

Post-irradiated carotid blowout syndrome (PCBS) is a dreaded complication that is associated with head-and-neck cancers (HNC) and its treatment [1–5]. CBS tends to occur in patients with HNC and those with radiation-induced necrosis, recurrent tumors, wound complications, or pharyngocutaneous fistulas [6–8]. Emergent surgical intervention in a hostile neck can result in significant mortality and morbidity rates [9, 10]. Endovascular therapy has been shown to improve patient outcomes [2, 11].

The reported outcomes of endovascular management of PCBS have varied considerably because of the diverse associated endovascular methods, location of the pathological lesions, disease processes and clinical severities [2, 7, 9, 11–14]. Various endovascular treatment algorithms have been proposed but none have been standardized [13, 15]. Although a reconstructive method with stent-graft has been reported to be a temporary method of management, recent reported series have shown that this method can yield promising results [14, 16–18]. Clinical severity has been reported to influence the rebleeding rate of patients with PCBS, but this has not widely validated [14, 19]. In this retrospective study, we evaluated the clinical and technical factors associated with the technical, hemostatic and survival outcomes of endovascular management of PCBS in the HNC patients. We also access the factors related to the outcomes and propose an algorithm of endovascular management of PCBS to highlight the decision of patient selection and post-procedural follow-up.

## Materials and Methods

### Patient Population

This retrospective study was reviewed and approved by an Institutional Review Board of Taipei Veterans General Hospital (No. 2014-08-004CC). Written consent for the endovascular procedure was obtained prior to intervention. Written informed consent was also given by the participants or their families for their clinical records to be used in this study. If the consent was not obtained, we made the patient records/information anonymized and de-identified prior to analysis according to the request of our IRB. Between 2000 and 2013, 96 patients with HNC complicated by PCBS who underwent endovascular treatment were included in this study (Table 1). They were initially classified into 2 groups according to the locations of pathological vascular lesions on angiogram. Group 1 comprised patients with the vascular lesion located at the branches of external carotid artery (ECA). Group 2 comprised patients with the vascular lesion located at the trunk of carotid artery, such as in the internal carotid artery (ICA), carotid bifurcation (CBF) and common carotid artery (CCA). Group 2 was further classified as group 2A and 2B, according to the method of endovascular management. The patients in group 2A accepted embolization (EM). The patients in group 2B accepted reconstructive management (RE).

**Table 1:** Demographic features of 96 patients of head and neck cancers with post-irradiated carotid blowout syndrome accepted endovascular management.

Factors	Group 1 (Branch, n = 40)	Group 2 (Trunk, n = 56)		Total (n = 96)
		2A (EM, n = 38)	2B (RE, n = 18)	
**Age**	53.8±12.1(28–80)	55.4±10.4(40–90)	49.9±7.3(34–65)	53.7±10.7(28–80)
**Sex (M/F)**	39/1	29/9	18/0	86/10
**Initial clinical Diagnosis (location)**				
Nasopharynx (upper)	11(27.5%)	16(42.1%)	1(5.5%)	28/96(29.2%)
Oropharynx (middle)	20(50.0%)	14(36.8%)	6(33.4%)	40/96(41.7%)
Hypopharynx/larynx (lower)	8(20.0%)	5(13.2%)	10(55.6%)	23/96(24.0%)
Others	1(2.5%)	3(7.9%)	1(5.5%)	5/96(5.1%)
**Interval of clinical diagnosis and onset of PCBS (year)**	1.6±0.8 (0.2–20)	11.0±2.5 (0.3–34)	3.8±3.0 (0.5–12)	3.5±2.8 (0.2–34)
**Re-irradiation for recurrent/secondary tumors**	15(37.5%)	19(50%)	12(66.7%)	45(47.9%)
**Periprocedural clinical findings**				
Necrotic tumor	33(82.5%)	28(73.7%)	15(83.3%)	76 (79.2%)
External skin wound	18(45.0%)	14(36.8%)	11(61.6%)	43 (44.8%)
Internal mucosal ulceration	34(85.0%)	27(71.1%)	3(16.7%)	64 (66.7%)
Pharyngocutaneous fistula	15(37.5%)	9(23.7%)	5(27.8%)	29 (30.2%)
**Clinical severity of PCBS**				
Ongoing	19(47.5%)	19(50.0%)	11(61.1%)	49 (51.0%)
Acute	21(52.5%)	19(50.0%)	7(38.9%)	47 (49.0%)
**Angiographic severity**				
Normal/irregularity	11(27.5%)	10(26.3%)	6(33.3%)	27 (28.1%)
Pseudoaneurysm /extravasation	29(72.5%)	28(73.7%)	12(66.7%)	69 (71.9%)
**Location of pathological lesion**				
ECA	40(100%)			
ICA		19(50.0%)	3(16.7%)	62 (64.6%)
CBF		16(42.1%)	7(38.9%)	26 (27.1%)
CCA		3(7.9%)	8(44.4%)	8(8.3%)
**Reintervention**	3(7.5%)	2(5.3%)	4(22.2%)	9(9.4%)

EM = embolization; RE = reconstructive management or stent-graft placement.

PCBS = post-irradiated carotid blowout syndrome.

All of the patients had undergone radiation therapy and presented with various degrees of soft tissue lesions and/or recurrent tumors in the head-and-neck region (Table 1). PCBS was classified into 2 types based on clinical emergency and severity, acute and ongoing [2, 9, 14]. The former group consisted of patients who suffered a clinical emergency, such as uncontrolled bleeding, and whose vital signs may have been unstable. The latter group consisted of patients whose status was nonemergent, including those who had controllable bleeding episodes. We compared these clinical severities with the angiographic severities and the outcomes.

### Interventional Procedures

#### Angiographic Evaluation

We used a transfemoral arterial approach to obtain a complete neuroangiogram of the supraaortic arteries. Based on the angiographic findings, the severity of the vascular injury was graded from 1 to 4 [7, 14, 20]. A grade of 1 indicated no angiographic vascular disruption. A grade of 2 was defined as focal irregularity of the diseased carotid artery. A grade of 3 was defined as a pseudoaneurysm of the injured carotid artery. A grade of 4 was defined as active extravasation from the ruptured artery. We further classified the angiographic severity into 2 categories, normal/irregularity (grade 1 and 2) and psudoaneurysm/extravasation (grade 3 and 4). The former category indicated that the diseased arterial wall was vulnerable to adjacent soft tissue process, but was still intact. The latter category indicated that the diseased artery had ruptured without a complete vascular wall. We compared these angiographic findings with the clinical severities.

### Endovascular Management

#### Embolization (EM)

**Fig 1:**
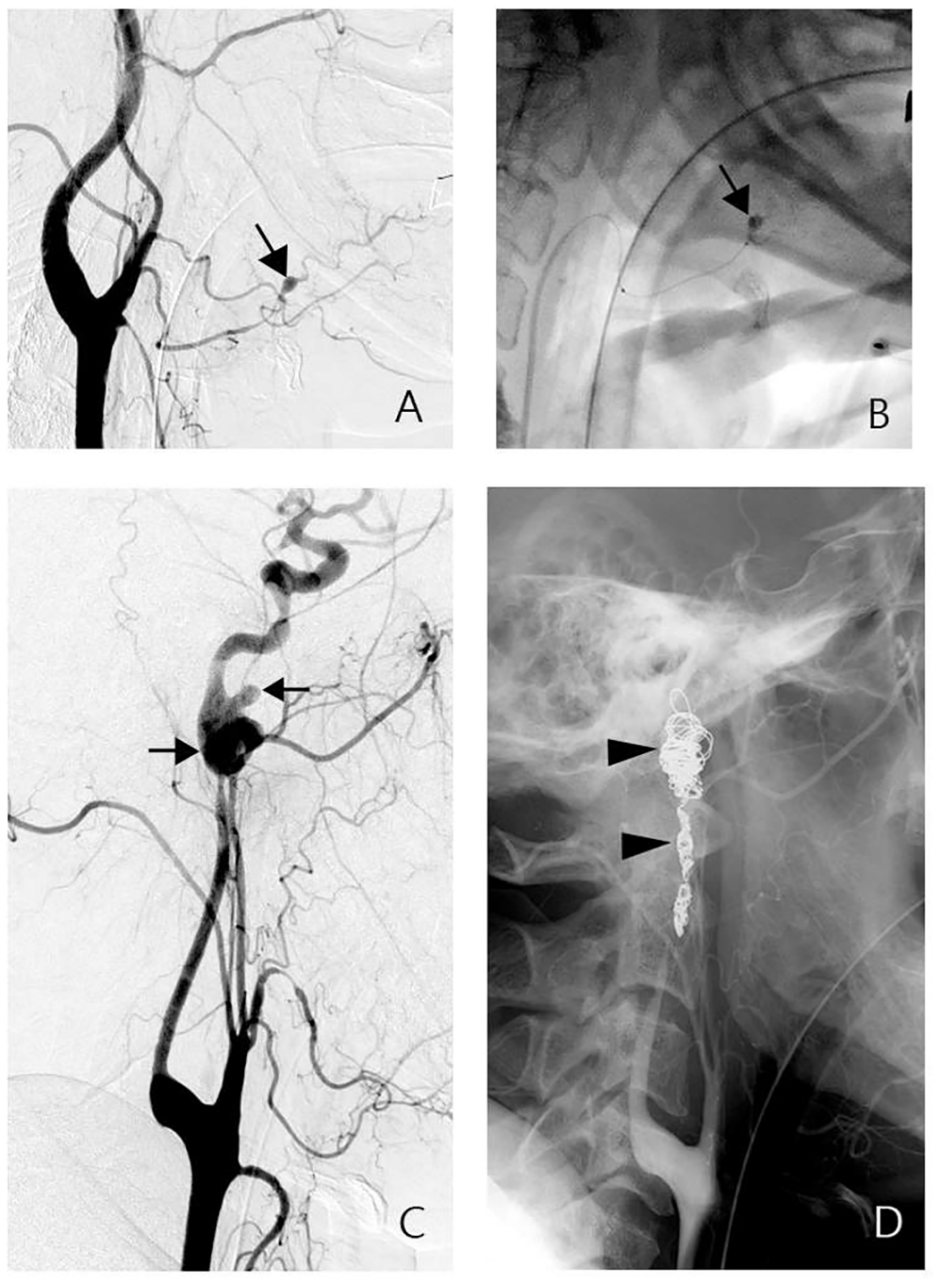
A-B, group 1 patient. Angiogram of the left carotid artery showing a pseudoaneurysm in the lingular artery (A, arrow). It was embolized by the injection of acrylic adhesive through a microcatheter (B, arrow). C-D, group 2A patient. Angiogram of the left carotid artery showing a complex pseudoaneurysm in the distal cervical ICA (C, arrows). Embolization of the vascular lesion and the ICA with fiber coils was noted in the control angiogram (D, arrowheads).

Group 1 and group 2A patients were treated by EM with permanent embolization of the pathological lesion and/or its parent artery [7, 14]. In group 1 patients, we injected microparticles (Embosphere, Biosphere Medical, Rockland, MA; polyvinyl alcohol [Ivalon], Laboratories Nycomed S.A., Paris, France) through the microcatheter to embolize angiographic category 1 lesions and the parent artery. For angiographic category 2 lesions, we deployed microcoils (Target Therapeutics, Fremont, CA) and/or injected acrylic adhesive (Histoacryl, Braun, Germany) to occlude the pathological lesion and its parent artery (Fig 1A and 1B).

For group 2A patients, a balloon test occlusion was attempted if the patient was hemodynamically stable and was cooperative. If the patients passed the occlusion test, we deployed the detachable balloons, microcoils and/or injected acrylic adhesive to occlude the pathologic lesion and its adjacent carotid artery (Fig 1C and 1D). Successful DE of the group 1 and 2A was defined as complete obliteration of the pathologic lesion and the parent carotid artery on control angiogram and the achievement of clinical hemostasis.

#### Reconstructive Management (RE, stent-graft placement)

**Fig 2:**
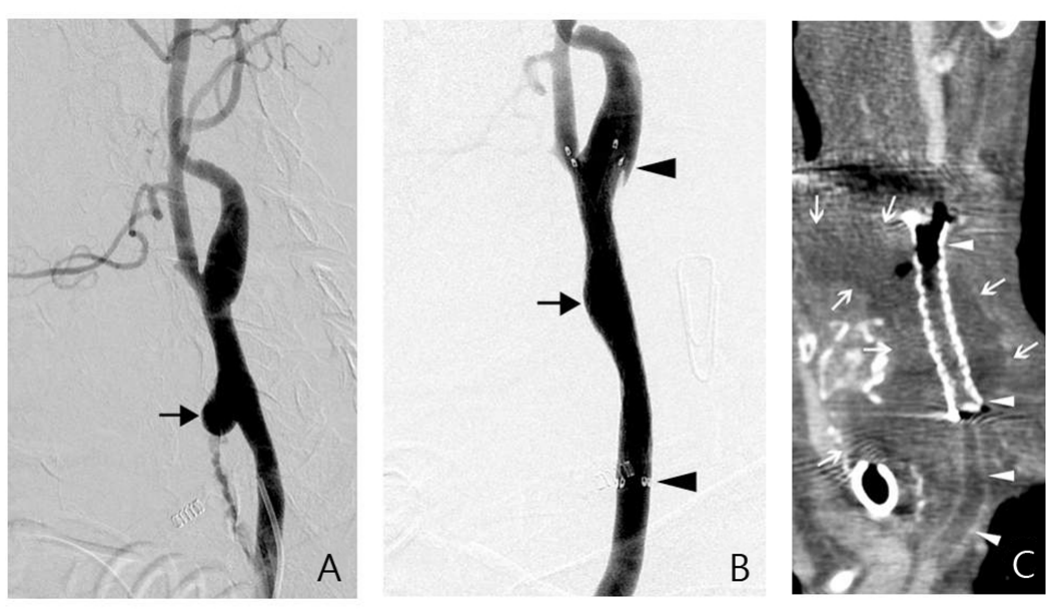
A-C, group 2B patient. Active extravasation from the pseudoaneurysm of left distal CCA was noted (E, arrow). We deployed a 10x60 mm Fluency stent-graft in the CCA (F, arrowheads) and achieved good coverage of the bleeding lesion (F, arrow). CT of the neck 6 months after curved multi-planar reconstruction of left carotid artery, showing asymptomatic septic thrombosis of the stent-graft and the carotid artery (G, arrowheads). The stent-graft was surrounded by a necrotic soft tissue lesion with abscess formation (G, arrows).

The indications for RE of group 2B patients were the patients at risk of permanent carotid occlusion, such as those with contralateral carotid occlusion, intolerance to a balloon occlusion test, or emergency status of the patient precluding an occlusion test [13, 14]. The technique of RE has been described in previous studies [14, 16]. We used a self-expandable stent-graft, including Wallgraft stent-graft (Boston Scientific Corp, Natick, Mass), Fluency stent-graft (Bard/Angiomed GmbH & Co, Karlsruhe, Germany), or Viabahn (W.L. Gore & Associates, Flagstaff, AZ, USA) (Fig 2A and 2B). Successful RE of group 2B was defined when adequate coverage of the pathologic lesion by stent graft with obliteration of the pathological vascular lesion on angiogram and clinical hemostasis was reached.

### Outcome Evaluation of Endovascular Procedures

Technical success and safety was evaluated immediately after the procedures. Clinical and/or imaging follow-up was performed every 3 months. If rebleeding occurred, emergency angiography and interventional management were performed. All of the patients had accepted adjuvant therapies for the necrotic tumors or soft tissue lesions, including chemoradiotherapy, skin graft surgery, aggressive wound care or hyperbaric oxygen therapy. If the tumor and/or the soft tissue lesion showed regressive or curative change, we classified it as resolution/regression of clinical disease. The other patients without clinical improvement were classified as having persistence/progression of clinical disease. The post-procedural clinical diseases were recorded as predictors of outcomes.

Three categories were applied to evaluate the outcomes of endovascular management, technical, hemostatic and survival. Technical outcomes were analyzed with the technical success (immediate hemostasis) and complications. Rebleeding and hemostasis time were examined as hemostatic outcomes. The hemostatic time refers to the period after the successful intervention and the presence of rebleeding or the follow-up period of this study. Survival outcomes included whether the patients were alive, had died, and if the latter, their survival time. The survival time refers to the period between the patients accepted endovascular management and their death or the follow-up period of this study.

### Statistical Analysis

Technical, hemostatic and survival outcomes were examined by the t-test, chi-squared test or Fisher’s exact test when appropriate. Endovascular methods, clinical severities, and the post-procedural clinical diseases were examined as predictors of the therapeutic outcomes. The clinical severity of PCBS was correlated with the angiographic severity using Fisher’s exact test. For all analyses, *P* < 0.05 was considered statistically significant.

## Results

### Baseline Characteristics

Table 1 summarizes the demographic features of all patients. Male predominance was noted in all patients. The location of the initially diagnosed cancers was most commonly at the oropharynx. The 5 patients had diagnoses of other cancers, including 1 lymphoma, 1 sarcoma and 3 metastatic carcinomas. All of the patients had at least one predisposing factor as their peri-procedural clinical findings. Reintervention of rebleeding was performed in 9 (9.4%) patients. The origin of this rebleeding from a new lesion of the branches of ECA were noted in 6 patients, including 3 of group 1, 2 of group 2A and 1 of group 2B. The other 3 patients of group 2B who accepted reintervention and included 1 case of type III endoleak of the stent-graft by an overlapped stent and 2 cases of progressive disease with new lesions in the margin of the stent-grafts. Regressive change of the post-procedural clinical disease was noted in 21 of the 96 patients (21.9%). They included tumor control by chemoradiotherapy or surgery in 7 patients and management of the soft tissue lesion by skin graft surgery or clinical wound care in 14 patients.

### Clinical and Angiographic Severity

For all patients, clinical severity correlated well with angiographic severity (*P*<0.0001, Table 1). In the analysis of the pathological locations of the group 1 and group 2, clinical severity also correlated well with angiographic severity (*P* = 0.0491 and *P* = 0.0001, respectively).

### Statistical Analysis of Outcomes of Endovascular Management

The outcomes related to the location of pathological lesion and endovascular methods, the clinical severity, and the post-procedural clinical disease are shown in Table 2.

**Table 2:** Endovascular methods, clinical severity, postprocedural clinical disease and outcomes of 96 patients of head and neck cancers with post-irradiated carotid blowout syndrome accepted endovascular management.

Factors Factors	Technical		Hemostatic		Survival	
	Success	Complication	Rebleeding	Hemostatic period (mon)	Alive	Survival time (mon)
**Endovascular methods**						
Group 1 (Branch, n = 40)	40 (100%)	1 (2.5%)	14 (35.0%)	10.9±18.8 (0.1–80)	9 (22.5%)	12.2±20.1 (0.2–80)
Group 2A (EM, n = 38)	38 (100%)	9 (23.7%)	5 (13.2%)	7.6±14.6 (0.01–60)	11 (29.0%)	7.7±14.6 (0.07–60)
Group 2B (RE, n = 18)	18 (100%)	9 (50.0%)	7 (38.9%)	2.9±3.0 (0.07–12)	2 (11.1%)	11.4±25.2 (0.07–110)
Total (n = 96)	96 (100%)	19 (19.8%)	26 (27.1%)	8.1±34.7 (0.01–80)	22 (22.9%)	10.3±34.1 (0.07–110)
P*	1.0	0.0001	0.0435	0.2367	0.7440	0.1392
P**	1.0	0.0052	0.0247	0.1343	0.4716	0.0598
P***	1.0	0.0489	0.0284	0.9439	0.9292	0.1846
**Clinical Severity**						
Ongoing (n = 49)	49(100%)	4(8.2%)	8(16.3%)	11.0±19.7 (0.07–80)	11(22.5%)	11.5±19.6 (0.07–80)
Acute (n = 47)	47(100%)	15(31.9%)	18(38.3%)	5.1±8.6 (0.01–45)	11(23.4%)	9.0±19.1 (0.07–110)
*P*	1.0	0.0035	0.0155	0.0216	0.4159	0.0709
**Postprocedural clinical disease**						
Resolution/Regression (n = 21)	21(100%)	2(9.5%)	3(14.3%)	26.0±25.7 (0.07–80)	14 (66.7%)	34.1±30.6 (0.3–110)
Persistence/progression (n = 75)	75(100%)	17(22.7%)	23(30.7%)	3.1±3.8 (0.01–22)	8 (10.7%)	3.6±4.0 (0.07–22)
*P*	1	0.2289	0.1714	<0.0001	<0.0001	<0.0001

P* = comparison of group 1, group 2A and group 2B;

P** = group 1 vs 2A;

P*** = group 2A vs 2B.

#### Technical Outcomes

1. Technical success:
  Successful endovascular management with achievement of immediate hemostasis was accomplished in all 96 patients during the initial procedures (*P* = 1.0).
2. Technical complication:
  Technical complications were noted in 19 of the 96 patients (19.8%, Table 2). They are described in the Table 3. Group 1 had the significantly lowest technical complication of the 3 groups (group 1 vs 2A vs 2B, 1/40[2.5%] vs 9/38[23.7%] vs 9/18[50.0%], *P* = 0.0001). In the analysis of the technical safety of EM by location of the pathological lesion, the occurrence of technical complications in group 1 was significantly lower than that in group 2A (1/40[2.5%] vs 9/38[23.7%], *P* = 0.0052). With respect to the technical safety between EM and RE, group 2A had significantly lower technical complications than group 2B (9/38[23.7%] vs 9/18[50.0%], *P* = 0.0489). In the analysis of clinical severity, patients with acute PCBS had significantly higher technical complications than patients with ongoing PCBS (15/47[31.9%] vs 4/49[8.2%], *P* = 0.0035).

**Table 3:** Technical complications of the 19 patients of post-irradiated carotid blowout syndrome accepted endovascular management.

Group	Cases	Description
**Group 1 (n = 40)**	1 (2.5%)	Acute infarction (major stroke): reflux of acrylic adhesive from the lingulofacial trunk to ECA and ICA
**Group 2A (n = 38)**	4 (10.5%)	Acute cerebral ischemia (3 major stroke and 1 minor stroke): including 1 case of reflux of acrylic adhesive from proximal ECA to ICA with major stroke and 1 case of intraprocedural massive blood vomiting with choking and hypoxic encephalopathy
	3 (7.9%)	Delayed cerebral ischemia (1 TIA and 2 minor stroke)
	1 (2.6%)	Delayed brain abscess formation
	1 (2.6%)	Delayed dislodgement of the detachable balloon through a skin fistula
**Group 2B (n = 18)**	2 (11.1%)	Acute infarction: acute embolism and major stroke
	7 (38.9%)	Delayed stenosis/occlusion of stent-graft: including 4 marginal stenosis/occlusion (2 asymptomatic marginal stenosis, 2 occlusion with delayed major stroke), 3 delayed septic thrombosis (2 asymptomatic, 1 associated with brain abscesses)

ECA: external carotid artery; ICA: internal carotid artery.

#### Hemostatic Outcomes

1. Rebleeding:
  Rebleeding after the initial endovascular procedure was noted in 26 (27.1%) of the 96 patients. Group 2A had the lowest occurrence of rebleeding of the 3 groups (group 1 vs 2A vs 2B, 14/40[35.0%] vs 5/38[13.2%] vs 7/18[38.9%], *P* = 0.0435). In analysis of the location of pathological lesions of EM, the occurrence of rebleeding in group 1 was significantly lower than that of group 2A (14/40[35.0%] vs 5/38[13.2%], *P* = 0.0247). For the endovascular methods between EM and RE, group 2A showed significantly lower rebleeding rates than that of group 2B (5/38[13.2%] vs 7/18[38.9%], *P* = 0.0489, Table 2). In analysis of clinical severity, patients with acute PCBS had significantly higher rebleeding rates than that of patients with ongoing PCBS (18/47[38.3%] vs 8/49[16.3%], *P* = 0.0155).
2. Hemostatic period:
  The average hemostatic period of the 96 patients was 8.1±34.7 (0.01–80) months. In the analysis of clinical severity, patients with acute PCBS had a significantly shorter hemostatic period than that of patients with ongoing PCBS (11.0±19.7[0.07–80] vs 5.1±8.6[0.01–45] months, *P* = 0.0216). The patients with persistent post-procedural clinical disease had significantly shorter hemostatic periods than those with regressive change. (26.0±25.7[0.07–80] vs 3.1±3.8[0.01–22] months, *P*<0.0001).

#### Survival Outcomes

1. Alive:
  After the initial endovascular procedure, 22 (22.9%) of the 96 patients survived. In the analysis of clinical severity, patients with acute PCBS had insignificantly lower survival than those with ongoing PCBS (11/47[23.4%] vs 11/49[22.5%], *P* = 0.4159). The status of postprocedural clinical disease had a significant influence on the survival of the patients (progression vs regression, 8/75[10.7%] vs 14/21[66.7%], *P<*0.0001).
2. Survival time:
  The average survival time of the 96 patients was 10.3±34.1 (0.07–110) months. In the analysis of clinical severity, patients with acute PCBS had insignificantly shorter survival time than those with ongoing PCBS (9.0±19.1[0.07–110] vs 11.5±19.6[0.07–80] months, *P* = 0.0709). Patients with persistence/progression of postprocedural clinical disease had significantly shorter survival time than those with regressive change (3.6±4.0[0.07–22] vs 34.1±30.6[0.3–110] months, *P*<0.0001).

## Discussion

All our patients had at least one local soft tissue lesion or necrotic tumor as their peri-procedural clinical finding, which also implies their close relationship with the occurrence of PCBS (Table 1) [6, 7]. Soft tissue lesions are difficult to identify by angiogram [21]. Additionally, it is difficult to evaluate deeply located soft tissue lesions in HNC patients with trismus or previous surgical reconstruction by clinical examination. We thus favor CT/CTA of the head and neck region to provide a complete pre-procedural planning if the patient is in nonemergent status [7].

The clinical severity of CBS was classified into three groups (acute, impending and threatened) to guide the management [2, 9]. In this study, we suggest a modified classification of PCBS patients into two groups according their clinical severity and emergency, acute or ongoing. The former PCBS is a clinical emergency involving conditions such as uncontrollable bleeding or hypovolemic status. The latter PCBS is an ongoing disease process without emergent condition, which includes the previous impending and threatened CBS. This simple clinical classification could guide endovascular management because it correlated well with angiographic severity (Table 1) [7, 14]. This classification was also able to guide the therapeutic planning for PCBS. For patients with acute CBS, emergent endovascular management is the only preferred therapeutic method. The patients with ongoing PCBS can accept a thorough pretreatment evaluation, such as a CT/CTA of head and neck region or a complete balloon occlusion test. This evaluation provides evidence for the performance of a vascular bypass surgery before EM or a premedication of RE, which is helpful for reducing the risk of technical complications during subsequent endovascular management.

In the present study, group 1 patients had significantly lower technical complications, but higher rebleeding, than the patients in group 2A (Table 2). The low rate of technical complications in group 1 was related to the anatomical factor and the different endovascular techniques. For example, microparticle can be used as an embolic agent for treating group 1 patients with slight angiographic severity but is not suitable to the group 2A patients. The causes of the rate of high rebleeding of group 1 included the wide anatomical vascular territory of ECA and its good reconstituted collaterals in the head and neck region. Embolization of the pathological lesion and its parent artery of ECA can have also aggravated local tissue ischemia, which may limit the subsequent soft tissue healing [7]. For the 5 patients in group 1 and 2A with rebleeding that accepted reintervention, the pathological lesions originated from the other branches of ECA. This suggests the post-procedural follow-up after initial management should include the whole irradiated territory of ECA.

In our study, group 2B had a significantly higher rate of technical complications and rebleeding than group 2A. We thus favor using DE as the first-line of management of CBS if the patient can pass an occlusion test. The causes of the high rates of technical complications of RE included the risk of cerebral thromboembolism by the inadequate antiplatelet medication in emergent status, septic thrombosis of the stent-graft in a contaminated field and delayed marginal stenosis/occlusion of the carotid artery by the strong radial force of the stent-graft (Table 3; Fig 2C) [14]. In comparison with the permanent occlusion of the whole diseased carotid artery of EM, the hemostatic effect of RE was associated with the coverage range of the stent-graft. Progressive change of the pathological soft tissue lesion beyond the stent-graft might also be associated with recurrent marginal pseudoaneurysms and rebleeding. Despite the unfavorable conditions in the application of RE, we still suggest it is a valid management strategy in patients with acute PCBS at risk of permanent carotid occlusion. In our study, delayed cerebral ischemic insult was noted in 3 patients of EM, even they could pass a balloon occlusion test (Table 3). It also highlights that a tailored preprocedural evaluation of permanent carotid occlusion should be reassessed with a balloon occlusion test before the patients of ongoing PCBS accept EM [22, 23].

We found that patients with advanced clinical severity had significantly higher technical complications, higher rebleeding rate and shorter hemostatic period than those with lower clinical severity (Table 2). These results favor the early evaluation and intervention of patients with post-irradiated CBS to improve the outcomes. We suggest early intervention because of the following reasons: 1) Advanced clinical severity may be associated with poor peri-procedural clinical conditions (e.g., aspiration pneumonia or choking by the massive blood vomiting), which could impair the final clinical outcome even though hemostasis is achieved. 2) In patients with severe hypovolemic status with vascular collapse or under aggressive surgical compression of the bleeding source, the inconspicuous pathological lesions in the branches of ECA may be temporarily unrecognized. These unrecognized lesions could subsequently increase the risk of rebleeding. 3) For the group 2 patients, there is risk of uncontrollable bleeding when performing a complete balloon occlusion test under full heparinization in patients with active extravasation and hypovolemic status. The results of the occlusion test are especially inconclusive when the patients have unstable vital signs and impaired consciousness. In these cases, patients have to accept RE, which is associated with a higher rate of technical complications and rebleeding than EM. 4) In emergent status, a standard dual antiplatelet premedication for RE is usually not possible. Further studies investigating the early prediction of PCBS can help to improve the outcomes of endovascular management.

**Fig 3:**
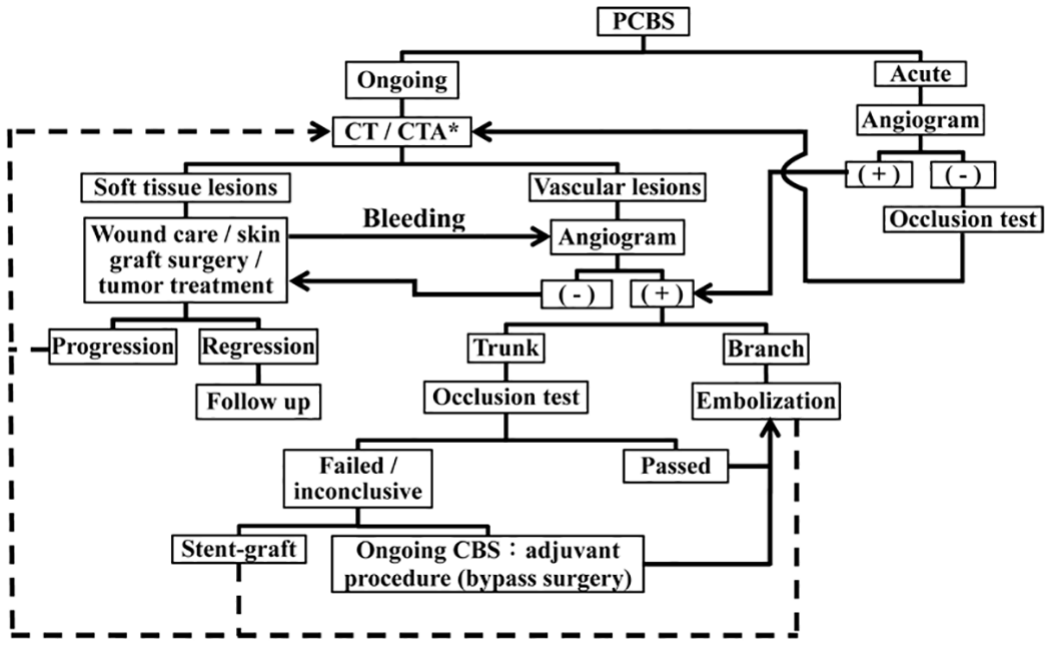
Algorithm of endovascular management of post-irradiated carotid blowout syndrome (PCBS). CT/CTA*: reference 7, 21. The soft tissue lesions include necrotic tumor, soft tissue wound and pharyngocutaneous fistula; the vascular lesions include pseudoaneurysm or extravasation. Dash line: indicates follow-up after the initial management.

We found that patients with resolution/regression of clinical disease had significantly longer hemostatic periods and better survival outcomes than those of persistence/progression of clinical disease (Table 2). In addition to prolonging life expectancy by cancer therapy, aggressive management of the tumor and/or soft tissue lesion of head and neck region can provide the following benefits to improve the survival outcomes of PCBS: 1. Approximately 80% blood supply of the wall of carotid artery comes from the adventia and the surrounding soft tissue [9]. Management of the adjacent soft tissue lesion can prevent rebleeding by restoring the blood supply to the diseased carotid arteries and controlling the disease progression to involve the other vascular territory. 2. The contaminated soft tissue lesions can progress to uncontrollable systemic infection or sepsis, which impair the patient’s survival. As the management of post-procedural clinical disease is related to the hemostatic and survival outcomes, we propose an algorithm to highlight the decision of patient selection and postprocedural follow-up (Fig 3). However, our findings in the patients’ survival are still minimal because of the limited numbers of the patients and the difficulty of management of the advanced clinical disease processes.

A limitation of this study was the short survival time, diverse disease course, and varied methods of previous treatment of these cancer patients, which made the long-term analysis difficult. Some patients who had rebleeding may chose non-aggressive care options or who had transitioned to hospice are not easily tracked. It was also difficult to quantitatively evaluate the poor quality of life of these HNC patients during their endovascular management. The analysis was further limited by the lack of a control group of clinical management to compare their outcomes. Further prospective research with controlled tumor staging and management is required to clarify the factors related to the outcomes of endovascular management of PCBS.

In summary, the endovascular management of PCBS of the patients of HNC has high technical success. The patients with bleeding lesions in the branches of the carotid artery had better technical safety but higher incidence of rebleeding than those with the lesions located in the trunk of the carotid artery. The patients accepting EM of PCBS were superior to those who had RE because of the significantly higher rates of technical complications and rebleeding in the latter group. We suggest taking embolization as a prior way of endovascular management in the patients of PCBS. We also suggest performing early endovascular intervention in patients with slight clinical severity and aggressive management of the post-procedural clinical disease to improve the outcomes.
